# Evaluation of the Mann Assessment of Swallowing Ability in Elderly Patients with Pneumonia

**DOI:** 10.14336/AD.2017.0102

**Published:** 2017-07-21

**Authors:** Yasuo Chojin, Tatsuji Kato, Mariko Rikihisa, Masami Omori, Shingo Noguchi, Kentaro Akata, Takaaki Ogoshi, Kazuhiro Yatera, Hiroshi Mukae

**Affiliations:** ^1^Department of Respiratory Medicine, Tobata Kyoritsu Hospital; ^2^Department of Rehabilitation, Tobata Kyoritsu Hospital; ^3^Department of Respiratory Medicine, University of Occupational and Environmental Health, Japan; ^4^Department of Respiratory Medicine, Wakamatsu Hospital of the University of Occupational and Environmental Health, Japan; ^5^Department of Respiratory Medicine, Unit of Translational Medicine, Nagasaki University Graduate School of Biomedical Sciences, Japan

**Keywords:** mann assessment of swallowing ability, elderly, aspiration risk, pneumonia, mortality, recurrence of pneumonia

## Abstract

Elderly pneumonia patients have various underlying diseases and social backgrounds, and it is difficult to predict their mortality using the current severity assessment tools. However, aspiration is a risk factor for mortality in pneumonia patients. In the evaluation of aspiration, endoscopic and video fluoroscopic methods are reliable but cannot be performed in all pneumonia patients. We evaluated the significance of the Mann Assessment of Swallowing Ability (MASA) in these patients. This study was prospectively performed between December 2014 and June 2015, and all adult hospitalized patients with pneumonia were consecutively enrolled. The MASA score was evaluated soon after admission. The outcome measures were in-hospital mortality, a recurrence of pneumonia within 30 days, 6-month mortality, and the detection of antibiotic-resistant bacteria. A total of 153 patients were ultimately included. The proportion of in-hospital mortality was greater among the severe MASA score patients than normal score patients (*p* < 0.01), as was the proportion of recurrence of pneumonia (*p* < 0.01) and 6-month mortality (*p* < 0.01). In addition, patients with a moderate MASA score more often experienced recurrence of pneumonia than normal score patients (*p* < 0.05). Furthermore, patients with a mild MASA score more often experienced recurrence of pneumonia (*p* < 0.01) and 6-month mortality (*p* < 0.05) than normal score patients. The areas under the curve were 0.74 (95% confidence interval [CI], 0.67-0.82) for in-hospital mortality, 0.75 (95% CI, 0.68-0.82) for recurrence of pneumonia, 0.72 (95% Cl, 0.64-0.81) for 6-month mortality, and 0.60 (95% CI, 0.46-0.73) for detection of antibiotic-resistant bacteria. A multivariate analysis showed an abnormal MASA score to be an independent risk factor for the recurrence of pneumonia (*p* = 0.001) and 6-month mortality (*p* = 0.005). The MASA is useful for predicting the mortality and recurrence of pneumonia in elderly patients.

Various severity assessment tools have been used to craft treatment strategies and predict mortality in the clinical practice of pneumonia, and the pneumonia severity index (PSI) and CURB-65 are commonly recommended in clinical practice [1,2]. However, elderly patients with pneumonia commonly have various factors that influence the treatment strategies and mortality, and the increasing number of elderly patients with pneumonia has hampered predicting mortality using these tools [3,4]. Furthermore, recent studies have underscored the importance of an evaluation of comorbid diseases and social background for predicting the mortality [5,6].

Comorbid diseases such as dementia and Parkinson’s disease often cause dysphagia, which is a major risk factor for aspiration pneumonia [7-9]. Elderly patients with pneumonia are often complicated with several of these diseases; therefore, the clinical influence of aspiration should be considered in elderly patients with pneumonia, even when obvious episodes of aspiration have not been demonstrated.

Endoscopic and video fluoroscopic methods have proven to be reliable in the evaluation of dysphagia [10], but these techniques cannot be performed in all patients with pneumonia, often due to a poor general condition or inadequate hospital facilities. The bedside water swallow test is known to identify many of those with aspiration [11], but it cannot be performed in all cases due to the risk of oropharyngeal aspiration and provides little information to guide treatment.

The Mann Assessment of Swallowing Ability (MASA) was created by Mann in 2002 as an assessment tool for identifying eating and swallowing disorders [12] and is used in patients with acute stroke [13]. Currently, the utility of the MASA has been reported in patients with various underlying diseases [14,15], and its high sensitivity and specificity for detecting aspiration has also been reported [16]. In addition, the MASA can quantify the aspiration risk via a bedside procedure and accounts for the cognitive function, which is a risk factor for aspiration pneumonia [17].

In the present study, we prospectively investigated the significance of assessing the MASA in patients with pneumonia.

## MATERIALS AND METHODS

### Study population

This study was prospectively conducted at Tobata Kyoritsu Hospital (218 beds in Kitakyushu City, Japan) between December 2014 and June 2015, and all adult hospitalized patients with pneumonia were consecutively enrolled. The following patients were excluded: (1) those younger than 20 years old; (2) those with esophageal cancer; and (3) those not evaluated using the MASA. This study was approved by the ethics committee of Tobata Kyoritsu Hospital (No. 15-01) and was registered in a clinical trial registry (UMIN 000016179). All patients provided their written informed consent.

### Definitions of pneumonia

Pneumonia was defined when the patient met all three of the following criteria: (1) at least one clinical symptom (fever, cough, sputum production, chest pain); (2) new infiltrates on chest radiography and/or computed tomography; and (3) a white blood cell count of ≥10,000/µl and/or increased levels of serum C-reactive protein. The definitions of community acquired pneumonia (CAP) and healthcare associated pneumonia (HCAP) were in accordance with the American Thoracic Society (ATS)/Infectious Disease Society of America (IDSA) guidelines [18].

### The Mann Assessment of Swallowing Ability

The MASA consists of 24 items, and each measured score is converted into a weighted 5 or 10 points [12], which are then summed to a 200-point maximum score. The total scores are then used to define four categories of aspiration risk, as follows: 170-200, no abnormality; 149-169, mild; 141-148, moderate; ≤140, severe. The MASA score was evaluated by expert speech-language-hearing therapists within three days after admission.

### Microbiological evaluation

Bacterial cultures were performed using a semi-quantitative method. The detection of bacteria was determined when the cultured bacterial volume from sputum samples was more than “2+” [19]. In addition, the presence of bacteria was determined when blood or pleural effusion cultures and/or urinary antigen tests for *Streptococcus pneumoniae* or *Legionella pneumophila* serogroup І (Binax^®^; Portland, ME, USA) were positive. In addition, methicillin-resistant *Staphylococcus aureus* (MRSA), *Pseudomonas aeruginosa*, extended spectrum β-lactamase (ESBL)-*Escherichia coli*, ESBL-*Klebsiella pneumoniae*, and *Acinetobacter* spp. were defined as antibiotic-resistant bacteria.

### Outcome measures

The major outcomes were in-hospital mortality, a recurrence of pneumonia within 30 days (defined as the emergence of new pneumonia after remission of the preceding pneumonia [20]), and 6-month mortality. In addition, the detection of antibiotic-resistant bacteria was also evaluated. The rates of recurrence of pneumonia and 6-month mortality were assessed by consulting the medical records and/or interview. Patients were treated by their attending physician, and the choice of antibiotics was entirely decided by the attending physician. Initial treatment failure was defined as death during initial antibiotic therapy or a change in the antibiotics due to insufficient response to the initially prescribed antibiotics at the judgement of the attending physician.

### Revised oral assessment guide

The revised oral assessment guide (ROAG) consists of eight items (voice, lips, mucus membranes, tongue, gums, teeth, saliva, and swallow), and each item is scored from 1 (healthy) to 3 (severe problems), with a possible total sum ranging from 8 to 24 points [21]. The evaluation of the ROAG was performed by expert dentists and dental hygienist.

### Statistical analyses

The STATA 14 software program (StataCorp LP, College Station, TX, USA) was used in this analysis, and Fisher’s exact test for tables (2 × 2) and the Mann-Whitney U test were applied. *P* < 0.05 was considered significant. The receiver operating characteristics (ROC) curves and areas under the curve (AUCs) were analyzed for each severity classification (normal, mild, moderate, and severe) according to the MASA score and each outcome. A univariate analysis was used to determine the ability of each variable to predict each outcome. A stepwise multiple logistic regression model was created for variables found to be significant (*p* < 0.05) in the univariate analysis to determine the variables with independent significant associations with each outcome.

**Figure 1.**
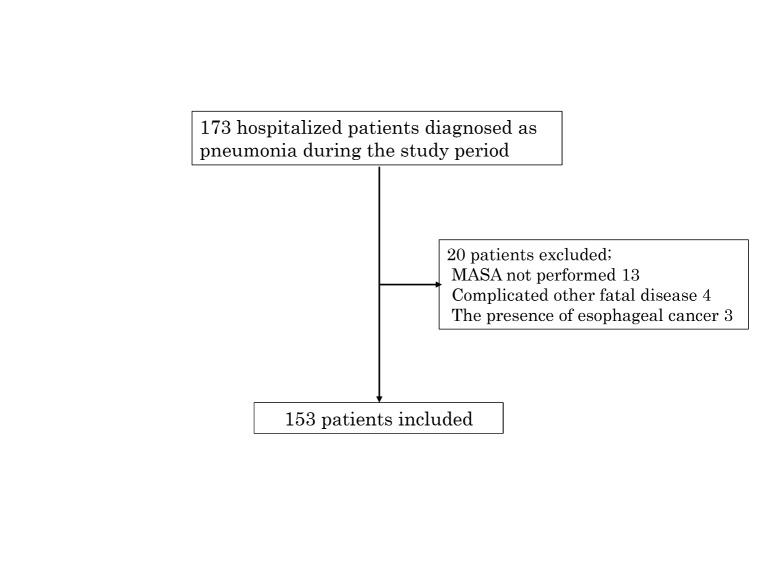
Flow chart.

## RESULTS

### Patient characteristics

A total of 173 hospitalized patients with pneumonia were enrolled, and 153 evaluated using the MASA were ultimately included in this analysis (Fig. 1). The clinical characteristics of these 153 patients are shown in Table 1. The average age was 85.4 ± 9.9 years old, and the ratio of females was 62.7%. In-hospital mortality was noted in 14 (9.2%) patients, and all 14 patients died because of pneumonia. Six-month mortality was noted in 29 (22.8%) patients, including 19 (65.5%) patients who died due to pneumonia and 10 patients who died because of sepsis (2 patients), COPD (1), liver dysfunction (1), caducity (1), and unknown causes (5). Recurrence of pneumonia within 30 days was observed in 47 (32.4%) patients. An abnormal MASA score was pointed out in 110 (71.9%) patients. In addition, abnormal respiration was pointed out in all patients (100%), followed by abnormal oral transit (86.9%), abnormal tongue strength (79.7%), and abnormal respiratory rate for swallowing (79.1%) in each assessment item of the MASA (Table 2).

### Pathogen distribution

The detected bacteria are listed in Table 3. Microbes were identified in 82 of 153 (53.6%) patients, and *S. pneumoniae* (14.6%) was the most commonly isolated, followed by *S. aureus* (13.4%), *Haemophilus influenzae* (13.4%), and *E. coli* (13.4%). The ratio of antibiotic-resistant bacteria was 19.5%.

**Table 1:** Characteristics of 153 patients with pneumonia.

Age (years), mean ± SD	85.4 ± 9.9
Female sex; n (%)	96 (62.7)
Performance status ≥3; n (%)	138 (90.2)
HCAP; n (%)	100 (65.4)
Comorbidity; n (%)	
Malignancy	38 (24.8)
Cerebrovascular disease	87 (56.9)
Laryngopharynx disorder	26 (17.0)
Chronic cardiovascular disease	69 (45.1)
Chronic respiratory disease	58 (37.9)
Chronic liver disease	6 (3.9)
Chronic kidney disease	8 (5.2)
Diabetes mellitus	23 (15.0)
Dementia	131 (85.6)
Clinical parameters; n (%)	
Orientation disturbance (confusion)	9 (5.9)
Respiratory failure	90 (58.8)
Systolic BP <90 mmHg or diastolic BP ≤60 mmHg	3 (2.0)
Body temperature ≤35 or ≥40 °C	8 (5.2)
Pulse rate ≥125 beats/min	17 (11.1)
Laboratory findings	
BUN ≥10.7 mmol/L	76 (49.7)
Glucose ≥13.9 mmol/L	7 (4.6)
Hematocrit <30%	23 (15.0)
Albumin <3.0 g/dl	52 (34.0)
PSI score; n (%)	
I-III	20 (13.1)
VI	65 (42.5)
V	68 (44.4)
Gastrogavage	5 (3.3)
ICU admission	13 (8.5)
Length of hospital stay (days)	19.1 ± 11.9
In-hospital mortality; n (%)	14 (9.2)
Recurrence of pneumonia within 30 days; n (%)^†1^	47 (32.4)
6-month mortality ^†2^	29 (22.8)

SD: standard deviation, HCAP: healthcare-associated pneumonia, BUN: blood urea nitrogen, PSI: pneumonia severity index, ICU: intensive care unit.

†1 Recurrence of pneumonia within 30 days was evaluated in 145 patients.

†2 Six-month mortality was evaluated in 127 patients.

**Table 2:** Number of abnormality of the clinical assessment items of the Mann Assessment of Swallowing Ability.

Clinical assessment items	Number of abnormality	
Alertness	69	(45.1)
Cooperation	95	(62.1)
Auditory comprehension	119	(77.8)
Respiration	153	(100)
Respiratory rate for swallowing	121	(79.1)
Dysphasia	83	(54.2)
Dyspraxia	41	(26.8)
Dysarthria	103	(67.3)
Saliva	34	(22.2)
Lip Seal	95	(62.1)
Tongue movement	105	(68.6)
Tongue strength	122	(79.7)
Tongue coordination	131	(85.6)
Oral preparation	86	(56.2)
Gag	116	(75.8)
Palate	87	(56.9)
Bolus clearance	85	(55.6)
Oral transit	133	(86.9)
Cough reflex	109	(71.2)
Voluntary cough	101	(66.0)
Voice	95	(62.1)
Trachea	1	(0.7)
Pharyngeal phase	103	(67.3)
Pharyngeal response	27	(17.6)

### In-hospital mortality according to the MASA

**Figure 2.**
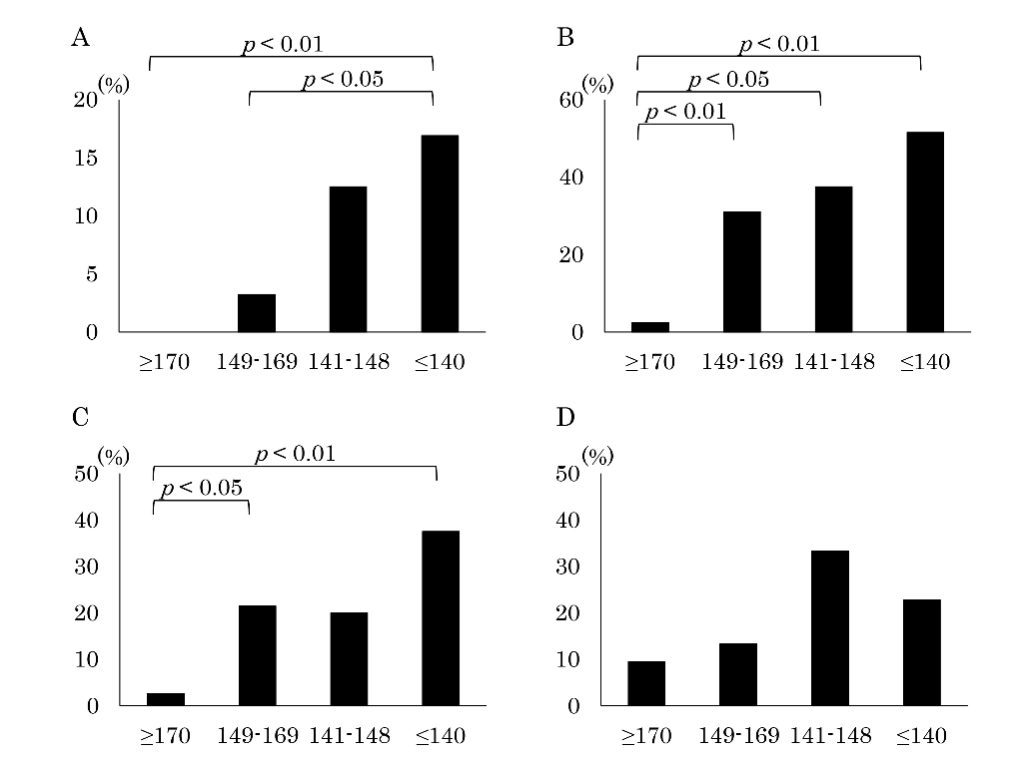
Prediction of each parameter according to the MASA score **A**) In-hospital mortality, **B**) Recurrence of pneumonia within 30 days, **C**) 6-month mortality, **D**) Detection of antibiotic-resistant bacteria.

All 153 patients were included in this analysis. Among the patients with normal, mild, moderate, and severe MASA scores, in-hospital mortality occurred in 0 of 43 (0.0%), 1 of 31 (3.2%), 1 of 8 (12.5%), and 12 of 71 (16.9%) patients, respectively (Fig. 2A). Patients with a severe score showed a significantly higher mortality rate than those with a normal score (*p* < 0.01). In addition, there were significant differences in the mortality rate between mild score and severe score patients (*p* < 0.05).

### Recurrence of pneumonia within 30 days according to the MASA

Six patients who did not admit remission of the first pneumonia and 2 lacking an outcome evaluation were not included in this analysis, leaving 145 patients for the evaluation. Among the patients with normal, mild, moderate, and severe MASA scores, the recurrence of pneumonia within 30 days occurred in 1 of 42 (2.4%), 9 of 29 (31.0%), 3 of 8 (37.5%), and 34 of 66 (51.5%) patients, respectively (Fig. 2B). The recurrence of pneumonia within 30 days differed significantly between those with normal scores and those with mild (*p* < 0.01), moderate (*p* < 0.05), and severe scores (*p* < 0.05).

**Table 3:** Microbes identified in patients with pneumonia.

*Staphylococcus aureus*	11	(13.4)
MSSA	5	(6.1)
MRSA	6	(7.3)
*Staphylococcus epidermidis*	1	(1.2)
*Streptococcus pneumoniae*	12	(14.6)
*Enterococcus* spp.	1	(1.2)
*Moraxella catarrhalis*	3	(3.7)
*Haemophilus influenzae*	11	(13.4)
BLNAR	6	(7.3)
*Escherichia coli*	11	(13.4)
ESBL	2	(2.4)
*Klebsiella pneumoniae*	7	(8.5)
ESBL	2	(2.4)
*Pseudomonas aeruginosa*	5	(6.1)
*Enterobacter* spp.	2	(2.4)
*Acinetobacter* spp.	1	(1.2)
*Legionella pneumophila*	1	(1.2)
Anaerobic bacteria	4	(4.9)
Oral bacteria	65	(79.3)
Others	3	(3.7)
Detected antibiotic-resistant bacteria^†^	16	(19.5)

Data are presented as n (%) unless otherwise stated. The percentages refer to 82 patients in whom some bacteria were identified.

MSSA: methicillin-sensitive *Staphylococcus aureu*s, MRSA: methicillin-resistant *Staphylococcus aureus,* BLNAR: β-lactamase non-producing ampicillin-resistant, ESBL: Extended spectrum β-lactamase

† MRSA, ESBL-producing bacteria, *Pseudomonas aeruginosa*, and *Acinetobacter* spp. were defined as antibiotic-resistant bacteria

### Six-month mortality according to the MASA

Fourteen patients who died during hospitalization and 12 lacking an outcome evaluation were not included in this analysis, leaving 127 patients for the evaluation. Among the patients with normal, mild, moderate, and severe MASA scores, 6-month mortalities occurred in 1 of 38 (2.6%), 6 of 28 (21.4%), 1 of 5 (20.0%), and 21 of 56 (37.5%) patients, respectively (Fig. 2C). Patients with a severe score showed a significantly higher mortality rate than those with a normal score (*p* < 0.01). In addition, there were also significant differences in the mortality rate between the mild score and normal score patients (*p* < 0.05).

### Detection of antibiotic-resistant bacteria according to the MASA

Eighty-two patients were included in this analysis. Among the patients with normal, mild, moderate, and severe MASA scores, antibiotic-resistant bacteria were detected in 2 of 20 (10.0%), 2 of 15 (13.3%), 1 of 3 (33.3%), and 10 of 44 (22.7%) patients, respectively (Fig. 2D). There were no marked differences in the detection rates based on MASA score.

### Prognostic accuracy for each outcome according to the MASA

**Figure 3.**
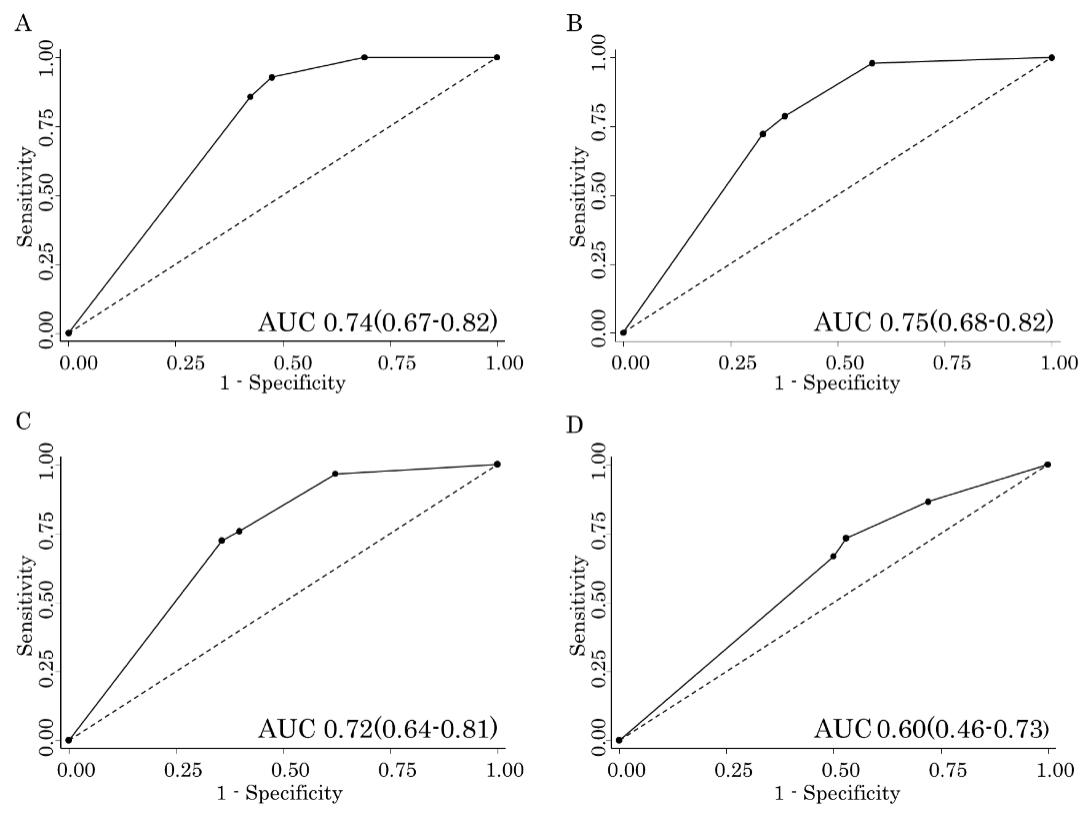
The receiver operating characteristics curve for each parameter according to the MASA score **A**) In-hospital mortality, **B**) Recurrence of pneumonia within 30 days, **C**) 6-month mortality, **D**) Detection of antibiotic-resistant bacteria.

The ROC curve between the MASA score severity classification and each outcome is shown in Figure 3. The AUCs using the ROC curve were 0.74 (95% confidence interval [CI], 0.67-0.82), 0.75 (95% CI, 0.68-0.82), 0.72 (95% Cl, 0.64-0.81), and 0.60 (95% CI, 0.46-0.73) for the in-hospital mortality, recurrence of pneumonia within 30 days, 6-month mortality, and the detection of antibiotic-resistant bacteria, respectively.

### Multivariate analysis of risk factors for in-hospital mortality

According to a univariate analysis, the significant risk factors for in-hospital mortality were cerebrovascular disease (*p* = 0.037), orientation disturbance (*p* = 0.02), hematocrit ≤30% (*p* = 0.031), and serum albumin ≤3.0 g/dl (*p* = 0.018), with no significance detected for MASA score (Supplmental Table 1). According to a multivariate analysis of these 4 items, the significant risk factors for in-hospital mortality were cerebrovascular disease (*p* = 0.024) and orientation disturbance (*p* = 0.020) (Table 4A).

### Multivariate analysis of risk factors for the recurrence of pneumonia within 30 days

A univariate analysis showed the MASA score (≤169) (*p* = 0.001) to be a significant risk factor for the recurrence of pneumonia within 30 days, in addition to cerebrovascular disease (*p* = 0.032), dementia (*p* = 0.028), inappropriate initial antibiotics (*p* = 0.034), and serum albumin ≤3.0 g/dl (*p* = 0.023) (Supplmental Table 2). A multivariate analysis of these 5 items showed only the MASA score (*p* = 0.001) to be an independent risk factor for the recurrence of pneumonia within 30 days (Table 4B).

### Multivariate analysis of risk factors for six-month mortality

A univariate analysis showed the MASA score (≤169) (*p* = 0.006) to be a significant risk factor for 6-month mortality, in addition to female sex (*p* = 0.031), chronic liver disease (*p* = 0.023), inappropriate initial antibiotics (*p* = 0.005), and serum albumin ≤3.0 g/dl (*p* ≤ 0.001) (Supplmental Table 3). A multivariate analysis of these 5 items showed the MASA score (*p* = 0.005) to be an independent risk factor for 6-month mortality, in addition to female sex (*p* = 0.025), inappropriate initial antibiotics (*p* = 0.037), and albumin ≤3.0 g/dl (*p* = 0.001) (Table 4C).

**Figure 4.**
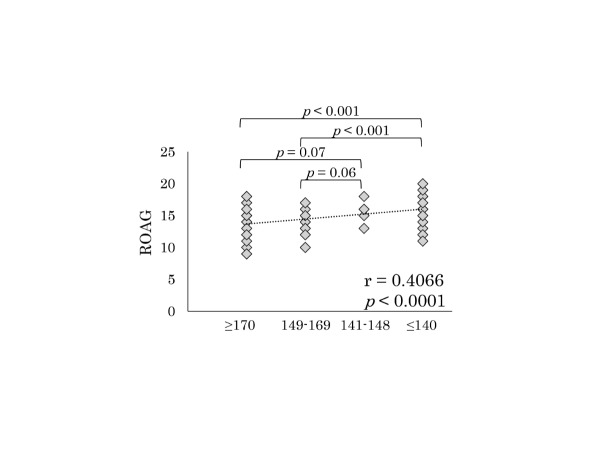
The revised oral assessment guide according to MASA

**Table 4:** Multivariate analysis of risk factors for each outcome

A. In-hospital mortality			
	Odds ratio	95% Confidence interval	*p* value
Cerebrovascular disease	7.48	1.31-42.84	0.024
Orientation disturbance	11.04	1.46-83.66	0.020
Hematocrit <30%	3.64	0.93-14.18	0.063
Albumin <3.0 g/dl	2.67	0.77-9.24	0.121

B. Recurrence of pneumonia within 30 days			
	Odds ratio	95% Confidence interval	*p* value
MASA score ≤169	36.20	4.17-314.67	0.001
Cerebrovascular disease	1.93	0.84-4.40	0.119
Dementia	0.79	0.12-5.12	0.808
Inappropriate initial antibiotics	2.40	0.98-5.86	0.055
Albumin <3.0 g/dl	2.09	0.90-4.84	0.085

C. Six-month mortality			
	Odds ratio	95% Confidence interval	*p* value
Female	0.30	0.10-0.86	0.025
MASA score =169	21.29	2.49-182.22	0.005
Chronic liver disease	10.49	0.94-117.07	0.056
Inappropriate initial antibiotics	3.25	1.07-9.86	0.037
Albumin <3.0 g/dl	5.83	2.02-16.87	0.001

### Correlation between the MASA and ROAG

The ROAG score was significantly higher in patients with a severe MASA score than in those with normal and mild scores (Fig. 4). In addition, there was a significant but weak correlation (*r* = 0.41, *p* < 0.0001) between the MASA score and ROAG.

## DISCUSSION

The present study prospectively analyzed the usefulness of an aspiration risk evaluation according to the MASA score for predicting the mortality and recurrence of pneumonia in patients with pneumonia. We first demonstrated that the MASA can be useful for predicting the mortality and the recurrence of pneumonia in clinical practice.

In this study, we showed that the MASA score was not an independent risk factor for in-hospital mortality but was an independent risk factor for 6-month mortality in a multivariate analysis (Table 4). Recently, Komiya et al. reported that aspiration pneumonia was related to the short- and long-term mortality; however, in their systematic review of aspiration risks, the results in each study varied widely with respect to the short-term mortality, which was strongly influenced by the differences in the study size, population and the definition of aspiration pneumonia [22]. While the MASA is known to be a useful tool for predicting aspiration in elderly people, there have been no reports of the usefulness of the MASA in patients with pneumonia [15, 16]. The results of this study suggest that the MASA can identify aspiration risks in patients with pneumonia and help predict the mortality of patients with pneumonia, although further investigations are needed with respect to the short-term mortality. Whereas, pneumonia was not the only cause of long-term mortality in this study. Repetitive aspiration pneumonia is usually related to a reduction in physical activity and malnutrition, and the long-term mortality in patients with aspiration pneumonia is influenced by various host factors [23]. Therefore, while not all deaths in this study were due to pneumonia, we nevertheless believe that the MASA is a useful tool for predicting the mortality in clinical practice, although whether the MASA can predict the long-term mortality due to pneumonia remains controversial.

We clarified in our study that an abnormal MASA score was an independent risk factor for predicting not only the mortality but also the recurrence of pneumonia. Generally, it is known that recurrence of pneumonia is not uncommon in elderly patients, and the presence of aspiration is an independent risk factor of recurrence of pneumonia and readmission due to pneumonia [24-26]. We think that patients with low MASA scores show a high aspiration risk, and similar to its utility in predicting mortality, the MASA can predict the recurrence of pneumonia.

In this study, we used all 24 items of the MASA scoring system, but the relative complexity of this system may be its weak point. Recent reports of the MASA have shown the usefulness of the modified MASA in various groups [14, 16, 27], and Ohira et al. reported that 17 of its 24 items are associated with aspiration risk [17]. However, according to our results, the abnormal items were necessarily inconsistent with their results; for example, “respiratory rate for swallowing” and “gag” were abnormal for almost 70-80% cases, although these items were not deemed significant for aspiration in the study of Ohira et al. [16]. Therefore, we need to investigate how the items are associated with predicting the mortality and recurrence in the patients with pneumonia.

Aspiration is a risk factor for detecting antibiotic-resistant bacteria [26, 28]. In this regard, the MASA score did not have strong power for detecting antidrug-resistant bacteria in this study. Whether antibiotic-resistant bacteria cultured in sputum are truly the pathogens of pneumonia remains controversial, and we also previously found that the MRSA relatively infrequently helped to determine the pathogenesis of pneumonia using the molecular method [29, 30]. In addition, the mortality associated with pneumonia is reported to be primarily due to advanced age and comorbidities and not an increased prevalence of resistant pathogens [23, 31]. Thus, the MASA might be a good assessment tool for predicting the mortality and recurrence of pneumonia without any influence on detecting antibiotic-resistant bacteria in cultivation.

Aside from the MASA, a female gender, oriental disturbance, cerebrovascular disease, chronic liver disease, dementia, a low level of serum albumin or hematocrit, and inappropriate initial antibiotics were also risk factors for mortality and the recurrence of pneumonia, according to a multivariate analysis. Regarding gender, it was reported that men are at a greater risk of aspiration pneumonia than women in systematic review, although various opinions have been reported with respect to gender [32]. The significant differences between genders might influence the aspiration risk, although whether the 6-month mortality was associated with aspiration was necessarily unclear. In contrast, age was not a significant risk factor for these three outcomes on a multivariate analysis in this study. Although age is generally thought to be a major risk factor for predicting mortality and/or recurrence of pneumonia [25, 33], the results of the present study might have been influenced by the higher rate of advanced-age patients than in other studies. Results similar to our observations were reported by Manabe et al., who found that age was not a significant risk factor for aspiration pneumonia among elderly patients [17].

Dysphagia is a common chronic problem with respect to older age, dementia, and chronic diseases, and the swallowing function has been reported to decline as the age increases, naturally leading to aspiration [34]. In addition, the recovery of dysphagia by swallowing rehabilitation alone might be difficult. However, the importance of oral care has been reported to help prevent the recurrence of pneumonia, thereby helping prevent repetitive pneumonia and reduce the mortality [35, 36]. The MASA score had a weak correlation with the ROAG, which is one of the evaluation of the oral function status [37, 38], in this study (Fig. 4). Therefore, we think that the MASA might be useful for suggesting an adequate time to start oral care in patients with pneumonia, thereby helping to reduce the rate of recurrence of pneumonia. In addition, it may be important for patients with low MASA scores to receive adequate evaluation and support in various aspects of their lives, such as meal contents, posture during meals, meal time, and care level [39]. Furthermore, pharyngeal electrical stimulation and clarithromycin administration might also be useful for treating patients with dysphagia [39, 40]. In this respect, further investigations are needed to clarify the usefulness of the MASA.

Several limitations associated with the present study warrant mention. First, although this study was a prospective study, it was a single-center trial. Second, we used the cut-off levels determined in acute stroke patients, but more optimal cut-off levels should be identified for predicting mortality in pneumonia patients in the future, such as the proposed optimal cut-off point (122 points) for predicting aspiration in dependent older adults [16]. Third, the MASA score might be influenced by the skills of each scorer and the timing of the examination, although the evaluation of MASA score was performed by only a few expert therapists at as early a stage of hospitalization as possible in the present study. Fourth, we cannot exclude the short-term influence of pneumonia itself for the MASA score because acute respiratory insufficiencies may lead to short-lived dysphagia. However, we believe that our results in this study are still significant for evaluating the usefulness of the MASA score at hospital admission in predicting the mortality and the recurrence of pneumonia in clinical practice.

In conclusion, the MASA is a useful tool for predicting the recurrence of pneumonia and the mortality in clinical practice of elderly people with pneumonia. However, further multicenter studies should be performed to confirm our results in patients with pneumonia, and easier assessment tools for assessing aspiration risks that can be widely used might still be needed, since the MASA is relatively complex.

**Suppmental Table 1:** A univariate analysis of the in-hospital mortality

	Odds ratio	95% Confidence interval	*p* value
Age ≥ 75 years	1.46	0.18-11.98	0.727
Age ≥ 85 years	2.47	0.66-9.27	0.179
Female sex	0.77	0.25-2.35	0.650
HCAP	0.68	0.22-2.08	0.500
MASA score ≤ 169	1.00		
Performance status ≥ 3	1.00		
MMSE ≤ 26	0.98	0.29-3.29	0.967
Neoplastic disease	0.81	0.21-3.07	0.757
Cerebrovascular disease	5.12	1.10-23.73	0.037
Gastroesophageal disorder	0.80	0.17-3.80	0.778
Chronic heart disease	1.24	0.41-3.73	0.699
Chronic respiratory disease	0.63	0.19-2.11	0.453
Chronic liver disease	1.00		
Chronic kidney disease	1.45	0.17-12.72	0.737
Collagen disease	10.61	0.63-179.80	0.102
Diabetes mellitus	0.94	0.20-4.49	0.935
Dementia	2.31	0.29-18.63	0.431
Sleeping medications	2.41	0.75-7.81	0.141
ACE inhibitor drugs	2.41	0.47-12.44	0.294
Inhaled corticosteroid	1.00		
Gastrogavage	2.60	0.27-25.0	0.409
Orientation disturbance	6.05	1.33-27.54	0.020
BUN ≥ 21 mg/dl	1.93	0.62-6.06	0.258
Respiratory rate ≥ 30 breaths/min	2.02	0.58-6.99	0.268
Systolic BP < 90 mmHg or diastolic BP ≤ 60 mmHg	1.00		
Body temperature ≥ 40 or ≤ 35 °C	1.45	0.17-12.7	0.737
Pulse rate ≥ 125 beats/min	1.38	0.28-6.76	0.693
SpO _2_ ≤ 90%, PaO _2_ ≤ 60 mmHg	1.84	0.55-6.17	0.321
Pleural effusion	1.92	0.63-5.90	0.253
Detected history of antibiotic-resistant bacteria	1.96	0.50-7.73	0.338
Inappropriate initial antibiotics	2.15	0.70-6.61	0.183
Antibiotic-resistant bacteria	2.14	0.48-9.49	0.315
Hematocrit < 30%	3.73	1.12-12.40	0.031
Albumin < 3.0 g/dl	4.02	1.27-12.70	0.018
Na < 130 mEq/ml	1.45	0.17-12.72	0.737
Glucose ≥ 250 mg/dl	4.47	0.78-25.52	0.092

HCAP: healthcare-associated pneumonia, MASA: The Mann Assessment of Swallowing Ability, MMSE: Mini Mental State Examination, ACE: angiotensin-converting enzyme, BUN: blood urea nitrogen, BP: blood pressure

**Suppmental Table 2:** A univariate analysis of recurrence of pneumonia within 30 days of admission

	Odds ratio	95% Confidence interval	*p* value
Age ≥ 75 years	1.85	0.49-7.00	0.362
Age ≥ 85 years	1.34	0.65-2.76	0.434
Female sex	0.58	0.28-1.17	0.129
HCAP	0.82	0.40-1.68	0.585
MASA score ≤ 169	33.09	4.38-249.78	0.001
Performance status ≥ 3	1.00		
MMSE ≤ 26	0.99	0.46-2.16	0.989
Neoplastic disease	1.25	0.56-2.75	0.585
Cerebrovascular disease	2.22	1.07-4.61	0.032
Gastroesophageal disorder	0.65	0.24-1.76	0.398
Chronic heart disease	0.52	0.25-1.06	0.072
Chronic respiratory disease	0.93	0.45-1.92	0.853
Chronic liver disease	4.47	0.79-25.31	0.091
Chronic kidney disease	2.95	0.63-13.73	0.169
Collagen disease	2.11	0.13-34.47	0.601
Diabetes mellitus	1.23	0.48-3.18	0.668
Dementia	5.41	1.20-24.31	0.028
Sleeping medications	1.46	0.62-3.42	0.389
ACE inhibitor drugs	0.89	0.22-3.59	0.866
Inhaled corticosteroid	1.00		
Gastrogavage	3.27	0.53-20.29	0.203
Orientation disturbance	1.27	0.29-5.55	0.752
BUN ≥ 21 mg/dl	0.84	0.42-1.70	0.635
Respiratory rate ≥ 30 breaths/min	0.78	0.30-2.02	0.705
Systolic BP < 90 mmHg or diastolic BP ≤ 60 mmHg	1.04	0.092-11.81	0.973
Body temperature ≥ 40 or ≤ 35 °C	0.33	0.039-2.85	0.316
Pulse rate ≥ 125 beats/min	0.85	0.28-2.58	0.779
SpO _2_ ≤ 90%, PaO _2_ ≤ 60 mmHg	1.44	0.70-2.94	0.320
Pleural effusion	1.17	0.54-2.53	0.681
Detected history of antibiotic-resistant bacteria	1.38	0.50-3.83	0.532
Inappropriate initial antibiotics	2.28	1.06-4.87	0.034
Antibiotic-resistant bacteria	0.99	0.77-1.25	0.904
Hematocrit < 30%	1.93	0.77-4.88	0.161
Albumin < 3.0 g/dl	2.31	1.12-4.77	0.023
Na < 130 mEq/ml	2.19	0.52-9.15	0.284
Glucose ≥ 250 mg/dl	1.00		

HCAP: healthcare-associated pneumonia, MASA: The Mann Assessment of Swallowing Ability, MMSE: Mini Mental State Examination, ACE: angiotensin-converting enzyme, BUN: blood urea nitrogen, BP: blood pressure

**Suppmental Table 3:** A univariate analysis of six-month mortality

	Odds ratio	95% Confidence interval	*p* value
Age ≥ 75 years	0.88	0.22-3.48	0.851
Age ≥ 85 years	2.05	0.83-5.08	0.120
Female sex	0.39	0.17-0.92	0.031
HCAP	1.13	0.46-2.75	0.791
MASA score ≤ 169	16.98	2.22-130.10	0.006
Performance status ≥ 3	1.00		
MMSE ≤ 26	0.85	0.34-2.08	0.715
Neoplastic disease	0.93	0.35-2.44	0.881
Cerebrovascular disease	2.05	0.85-4.94	0.111
Gastroesophageal disorder	0.35	0.08-1.63	0.182
Chronic heart disease	0.56	0.24-1.32	0.185
Chronic respiratory disease	1.22	0.52-2.83	0.651
Chronic liver disease	7.68	1.33-44.35	0.023
Chronic kidney disease	2.71	0.57-12.89	0.210
Collagen disease	1.00		
Diabetes mellitus	0.76	0.23-2.48	0.652
Dementia	5.06	0.64-40.06	0.125
Sleeping medications	1.41	0.52-3.81	0.494
ACE inhibitor drugs	0.40	0.05-3.35	0.400
Inhaled corticosteroid	0.59	0.12-2.81	0.504
Gastrogavage	1		
Orientation disturbance	1.00		
BUN ≥ 21 mg/dl	0.55	0.23-1.30	0.172
Respiratory rate ≥ 30 breaths/min	0.82	0.25-2.68	0.742
Systolic BP < 90 mmHg or diastolic BP ≤ 60 mmHg	1.00		
Body temperature ≥ 40 or ≤ 35 °C	1.00		
Pulse rate ≥ 125 beats/min	1.00		
SpO _2_ ≤ 90%, PaO _2_ ≤ 60 mmHg	0.85	0.37-1.96	0.701
Pleural effusion	1.69	0.71-4.06	0.238
Detected history of antibiotic-resistant bacteria	1.65	0.52-5.20	0.395
Inappropriate initial antibiotics	3.61	1.48-8.82	0.005
Antibiotic-resistant bacteria	3.41	0.88-13.25	0.077
Hematocrit < 30%	2.80	0.96-8.19	0.060
Albumin < 3.0 g/dl	4.89	2.03-11.78	≤0.001
Na < 130 mEq/ml	2.71	0.57-12.89	0.210
Glucose ≥ 250 mg/dl	0.84	0.09-7.82	0.878

HCAP: healthcare-associated pneumonia, MASA: The Mann Assessment of Swallowing Ability, MMSE: Mini Mental State Examination, ACE: angiotensin-converting enzyme, BUN: blood urea nitrogen, BP: blood pressure
